# Progress of isolation, chemical synthesis and biological activities of natural chalcones bearing 2-hydroxy-3-methyl-3-butenyl group

**DOI:** 10.3389/fchem.2022.964089

**Published:** 2022-08-15

**Authors:** Jiadai Zhai, Bingxia Sun, Feng Sang

**Affiliations:** ^1^ Research Center of Chemical Biology and Pharmaceutical Chemistry, School of Life Sciences and Medicine, Shandong University of Technology, Zibo, China; ^2^ School of Pharmaceutical Science and Technology, Tianjin University, Tianjin, China

**Keywords:** chalcone, 2-hydroxy-3-methyl-3-butenyl, isolation, synthesis, biological activity

## Abstract

Chalcones have a three-carbon α,β-unsaturated carbonyl system composed of two phenolic rings. Many chalcones have shown broad spectrum of biological activities with clinical potentials against various diseases. They are usually abundant in seeds, fruit skin, bark and flowers of most edible plants. Among them, chalcones bearing 2-hydroxy-3-methyl-3-butenyl (HMB) group have been reported several times in the past few decades due to their novel scaffolds and numerous interesting biological activities. In this paper, we reviewed the isolation of twelve natural chalcones and a natural chalcone-type compound bearing 2-hydroxy-3-methyl-3-butenyl group discovered so far, and reviewed their synthesis methods and biological activities reported in the literature. We anticipate that this review will inspire further research of natural chalcones.

## 1 Introduction

**FIGURE 1:**
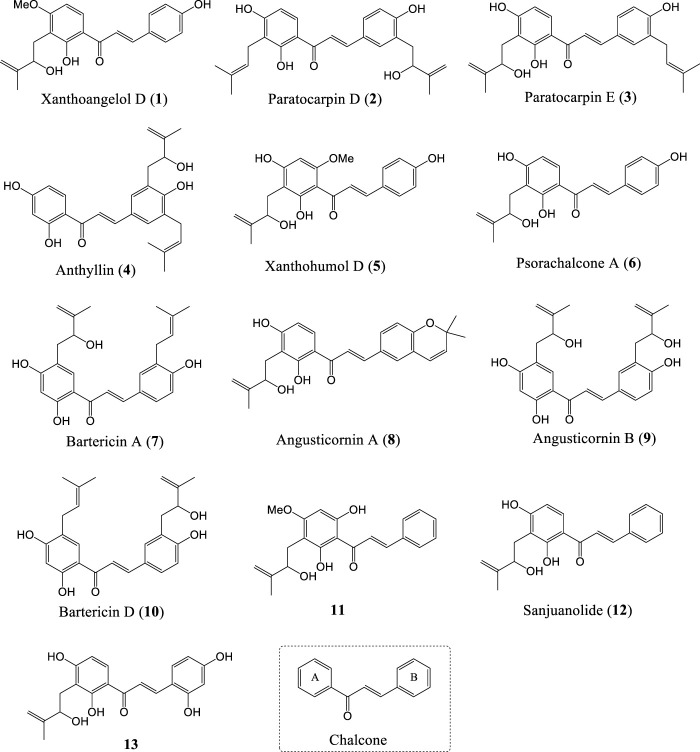
Structures of natural chalcones and a chalcone-type compound bearing HMB group (**1**-**13**) and chalcone.

The existing chalcones mainly include natural products and synthetic compounds (Rudrapal et al., 2021; Zhang et al., 2021; Yuan et al., 2022), and have been shown to exhibit a variety of biological activities, such as anticancer (Konieczny et al., 2007), anti-inflammatory (Nowakowska, 2007), antibacterial (Nielsen et al., 2005), antiviral (Duran et al., 2021), antimalaria (Smit and N’Da, 2014), and so on. It is an important approach for preclinical drug development to find new scaffolds from natural products and screen out lead compounds with high activity and low toxicity through chemical synthesis and structure-activity relationship study (SAR) (Gomes et al., 2017; Duvauchelle et al., 2021; Jasim et al., 2021; Knockleby et al., 2021). Chalcones have been extensively studied, and many reviews have been published in a wide variety of journals (Zhuang et al., 2017; Qin et al., 2020; Salehi et al., 2021). However, to our knowledge, there is no review of natural chalones bearing HMB group so far. Since the 1990s, twelve natural chalcones (1–7, 9–13) and a natural chalcone-type compound (8, Angusticornin A) with HMB group on A-ring or B-ring have been isolated and reported successively (Baba et al., 1990; Hano et al., 1995; Pistelli et al., 1996; Stevens et al., 2000; ElSohly et al., 2001; Ngameni et al., 2004; Ngadjui et al., 2005; RenQi and Shi, 2008; Shaffer et al., 2016; Yang and Jiang, 2021) (Figure 1). And the HMB group in their structures have also been proved to be an essential functional group for some biological activities (Sugii et al., 2005; Park et al., 2015). This review provides a research progress of the isolation, chemical synthesis and biological activities of natural chalcones bearing HMB group, and the plant species and biological activities of these chalcones are illustrated in Table 1.

**TABLE 1:** Plant species and biological activities of natural chalcones bearing HMB group.

Chalcones	Plant Species	Biological Activities	References
Xanthoangelol D (**1**)	*Angelica keiskei*	NF-κB inhibitory activity	Baba et al. (1990)
		Enzyme inhibitory activity	Sugii et al. (2005)
		Antiviral activity	Li et al. (2015)
			Park et al. (2015)
Paratocarpin D (**2**)	*Paratocarpus venenosa*	No cytotoxic or anti-inflammatory activity	Hano et al. (1995)
	*Adansonia digitata* L.		Liu et al. (2018)
			Ibraheem et al. (2021)
Paratocarpin E (**3**)	*Paratocarpus venenosa*	Cytotoxic activity	Hano et al. (1995)
	*Hedysarum gmelinii*	Anti-inflammatory activity	Liu et al. (2005)
	*Euphorbia humifusa*	Antibacterial activity	Gao et al. (2016)
			Liu et al. (2018)
			Li et al. (2019)
Anthyllin (**4**)	*Anthyllis hermanniae*	Unreported	Pistelli et al. (1996)
	*Humulus lupulus* cv.	Anti-inflammatory activity	Stevens et al. (2000)
	*Humulus lupulus* L.	Enzyme inhibitory activity	Zhao et al. (2003)
	*Humulus lupulus* L.	Enzyme inhibitory activity	Liu et al. (2005)
	*Humulus lupulus* L.	Antioxidant and cytotoxic activity	Choi et al. (2011)
Xanthohumol D (**5**)	*Humulus lupulus*	Enzyme inhibitory activity	Tronina et al. (2013)
		Anti-inflammatory activity	Yu et al. (2014)
	*Humulus lupulus* L.	Antibacterial activity	Sangiovanni et al. (2019)
			Fu et al. (2020)
Psorachalcone A (**6**)	*Maclura tinctoria* L.	Antifungal activity	ElSohly et al. (2001)
	*Psoralea corylifolia*	Enzyme inhibitory activity	Li et al. (2002)
	*Dorstenia angusticornis* and *Dorstenia barteri* var. *subtriangularis*	No antibacterial activity	Yin et al. (2004)
	*Morus nigra*	No cytotoxic activity	Ngadjui et al. (2005)
		Antibacterial activity	Zhai et al. (2019)
		No enzyme inhibitory activity	Li et al. (2019)
			Wang et al. (2021)
Bartericin A **(7)**	*Dorstenia barteri* var. *subtriangularis*	Antibacterial activity	Ngameni et al. (2004)
			Fu et al. (2020)
Bartericin D (**10**)	*Dorstenia barteri* var. *subtriangularis*	Unreported	Ngameni et al. (2004)
Angusticornin A (**8**)	*Dorstenia angusticornis* and *Dorstenia barteri* var. *subtriangularis*	Antibacterial activity	Ngadjui et al. (2005)
			Fu et al. (2020)
Angusticornin B (**9**)	*Dorstenia angusticornis* and *Dorstenia barteri* var. *subtriangularis*	Synergistic antibacterial activity	Ngadjui et al. (2005)
			Kuete et al. (2011)
chalcone **11**	*Anaphalis lactea*	No antibacterial activity	RenQi and Shi. (2008)
			Fu et al. (2020)
Sanjuanolide (**12**)	*Dalea frutescens*	Cytotoxic activity	Shaffer et al. (2016)
	*Artocarpus integer*	Cytotoxic activity	Zhai et al. (2019)
		Anti-inflammatory activity	Fang et al. (2019)
			Duong et al. (2021)
chalcone **13**	*Morus alba*	Antioxidant activity	Yang and Jiang. (2021)

## 2 Natural chalcones and a natural chalcone-type compound bearing HMB group

### 2.1 Xanthoangelol D (1)

#### 2.1.1 Isolation and biological activities

Xanthoangelol D and five other chalcones were extracted from fresh roots of *Angelica keiskei* collected in Hachijyo Island (Japan) by using ethyl acetate (Baba et al., 1990). Subsequently, the results of Sugii et al. showed that Xanthoangelol D suppresses basal and tumor necrosis factor-α-induced endothelin-1 (ET-1) production, by inhibiting the activation of nuclear factor-kappa B (NF-κB), therefore, may be useful for the treatment of diseases involved NF-κB activation (Sugii et al., 2005). Kil et al. also isolated Xanthoangelol D from the aerial parts of *Angelica keiskei* Koidzumi together with twelve other chalcones, and Xanthoangelol D did not exhibit significant activity in the assay of promoter activity on heat shock protein 25 (*hsp25*, murine form of human *hsp27*) (Kil et al., 2015). Xanthoangelol D showed strong potein tyrosine phosphatase 1B (PTP1B) inhibitory effect with IC_50_ value of 3.97 ± 0.37 μg/ml (Li et al., 2015). Interestingly, the inhibitory effects of Xanthoangelol D (substitution of A-ring with the HMB group) on severe acute respiratory syndrome coronavirus (SARS-CoV) chymotrypsin-like protease activity produced 4-fold (IC_50_ = 26.6 ± 5.2 μM) higher potency than analogue that substitution of A-ring with the 3-methyl-2-butenyl group (Park et al., 2015). Furthermore, Xanthoangelol D did not exhibit anti-platelet-activities *in vivo* according to Ohkura et al. (Ohkura et al., 2016).

#### 2.1.2 Chemical synthesis

**SCHEME 1:**
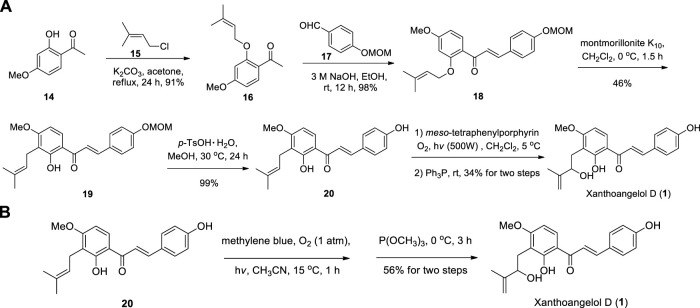
**(A)** Synthesis of Xanthoangelol D by Li et al.; **(B)** Synthesis of Xanthoangelol D by Sugamoto et al.

Li et al. reported the first synthesis of Xanthoangelol D with Schenck ene reaction using tetraphenylporphin (TPP) as the photosensitizer followed by reduction with triphenylphosphine (Li et al., 2019), and the key intermediate **20** can be obtained through Claisen-Schmidt condensation, [1,3]-sigmatropic rearrangement and deprotection by using the method of Sugamoto et al. (Sugamoto et al., 2008; Sugamoto et al., 2011) (Scheme 1A). One year later, Sugamoto et al. synthesized Xanthoangelol D in 56% yield by using the photooxygenation of prenylated chalcone **(20)** in the presence of methylene blue in acetonitrile followed by reduction with trimethylphosphite (Sugamoto et al., 2020) (Scheme 1B). These two methods described above provide important reference for the construction of HMB group in chalcone derivatives.

### 2.2 Paratocarpin D and E (2, 3)

#### 2.2.1 Isolation and biological activities

In 1995, Paratocarpin D and E were isolated from the Indonesian moraceous plant (Bark of *Paratocarpus venenosa* Zoll) by Hano et al. (1995) for the first time. Liu et al. (2005) also reported the isolation of Paratocarpin E along with two other new chalcones from the roots of *Hedysarum gmelinii* (collected from Inner Mongolia, China), and it was the first time that Paratocarpin E has been isolated from *Hedysarum* genus (Liu et al., 2005). Gao et al. isolated Paratocarpin E from *Euphorbia humifusa* Wild., and Paratocarpin E showed significant cytotoxicity against five cancer cell lines (MCF-7, 786-O, 769-P, U-937 and HL-60) with IC_50_ values ranging from 19.6 to 28.6 μM. According to the report, Paratocarpin E typical apoptosis of MCF-7 cells by activating p38 and JNK and inhibiting Erk pathway, and affect apoptosis and autophagy by promotes the activation and nuclear translocation of NF-κB (Gao et al., 2016; Al-Emam et al., 2019). Paratocarpin D and E were evaluated for antiproliferative activity against five human cancer cell lines (HepG2, A549, Du145, BGC823, and HCT116) and *in vitro* anti-inflammatory activity by Liu et al. (2005) but only Paratocarpin E exhibited weak inhibitory effects (IC_50_ values in the range of 10.33–18.18 μM) on lipopolysaccharide-induced nitric oxide production in murine microglial BV-2 cells (Liu et al., 2018). Furthermore, the racemate Paratocarpin E obtained by chemical synthesis exhibited significant antibacterial activity (MIC = 6.25 μg/ml) against *Bacillus subtilis* strain (Li et al., 2019). In 2021, Ibraheem et al. isolated Paratocarpin D from baobab (*Adansonia digitata* L.) fruit pulp methanolic extract, however, no activity data of individual compounds were reported (Ibraheem et al., 2021).

#### 2.2.2 Chemical synthesis

**SCHEME 2:**
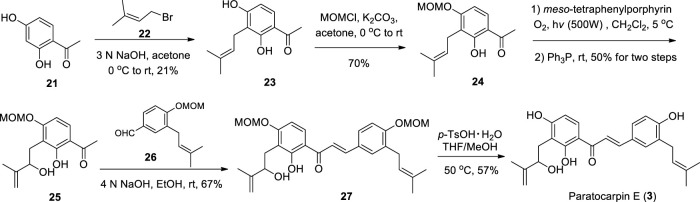
Synthesis of Paratocarpin E.

Li et al. reported the synthesis of Paratocarpin E using key intermediate **24** as the starting material, employing Schenck ene reaction, Claisen-Schmidt condensation and deprotection, respectively (Li et al., 2019) (Scheme 2). And the intermediate **24** can be prepared from 2,4-dihydroxyacetophenone **(21)** in two steps (Dong et al., 2007). However, the chemical synthesis of Paratocarpin D has not been reported yet.

### 2.3 Anthyllin (4)

In 1996, Anthyllin has been isolated from the aerial parts of *Anthyllis hermanniae*, along with six chalcone and isoflavonoid derivatives (Pistelli et al., 1996). Up to now, there have been no previous reports on biological activity or chemical synthesis of Anthyllin.

### 2.4 Xanthohumol D (5)

#### 2.4.1 Isolation and biological activities

In 2000, Xanthohumol D was isolated from *Humulus lupulus* cv. “Galena” by Stevens et al. for the first time (Stevens et al., 2000; Bocquet et al., 2018; Zhou et al., 2021). Zhao et al. isolated Xanthohumol D from the ethyl acetate fraction of *Humulus lupulus* L., and Xanthohumol D significantly inhibited NO production at 5 μg/ml (completely suppressed the expression of inducible NO synthase induced by lipopolysaccharide/IFN-γ) (Zhao et al., 2003; Zhao et al., 2005). In 2004, Chadwick et al. isolated Xanthohumol D from spent Nugget hop pellets (*Humulus lupulus* L. cv. Nugget) by supercritical CO_2_ extraction Chadwick et al. (2004). Subsequently, Xanthohumol D was tested for induction of quinone reductase in Heap 1c1c7 murine hepatoma cells by Liu et al. (2005) and the CD value was 7.4 ± 0.7 μM. Chesnokova et al. also isolated Xanthohumol D from hops (*Humulus lupulus*) by using Soxhlet apparatus Chesnokova et al. (2009). Choi et al. isolated Xanthohumol D from ethanolic extract of hops (*Humulus lupulus* L.), and Xanthohumol D was used to determine the inhibition of quinone reductase-2 (IC_50_ = 110 ± 27 μM) (Choi et al., 2011; Cieśla and Moaddel, 2016; Wei et al., 2016; Chen et al., 2018). Tronina et al. (2013) assessed the ability of Xanthohumol D to scavenge 2,2′-diphenyl-1-picrylhydrazyl (DPPH) radicals (IC_50_ = 2.37 ± 0.40 μM). In addition, the antiproliferative activity of Xanthohumol D against MCF-7 (IC_50_ = 20.60 ± 0.22 μM), PC-3 (IC_50_ = 37.88 ± 13.90 μM) and HT-29 (IC_50_ = 78.33 ± 8.83 μM) human cancer cell lines were also determined. Yu et al. (2014) isolated Xanthohumol D and seven other chalcones from *Humulus lupulus*, and the quinone reductase induction activity results showed that Xanthohumol D has moderate activity (IR = 2.22 ± 0.05, viability = 0.45%) in using human Heap 1c1c7 cells at the concentration of 20 μM. Sangiovanni et al. (2019) isolated Xanthohumol D from hop extracts (*Humulus lupulus* L. cultivar Cascade), and reported the anti-inflammatory activity of the hop extracts (Xanthoangelol D and Xanthoangelol A as the main active components) in human gastric epithelial cells. Fu et al. (2020) evaluated the antibacterial activities of synthetic Xanthohumol D against two Gram positive bacteria (*Staphylococcus aureus* CMCC 26003, *Bacillus subtilis* CMCC(B) 63,501) and two Gram negative bacteria (*Escherichia coli* CMCC 44102, *Pseudomonas aeruginosa* CMCC (B) 10,104), and Xanthohumol D showed significant activity towards *Bacillus subtilis* (MIC = 12.5 μg/ml) but no obvious inhibitory activity to the other three strains (MIC > 200 μg/ml).

#### 2.4.2 Chemical synthesis

**SCHEME 3:**
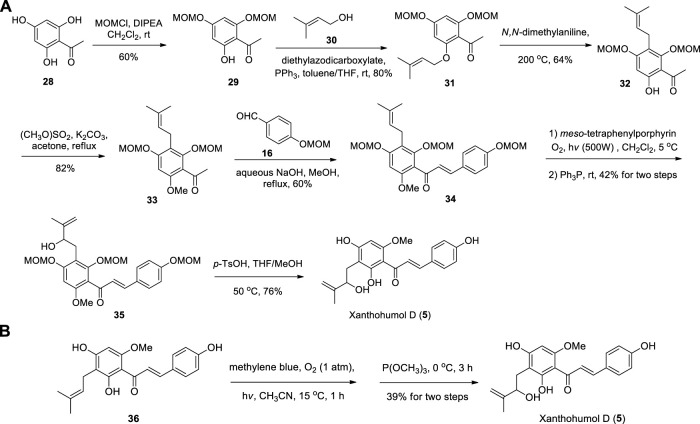
**(A)** Synthesis of Xanthohumol D by Fu et al.; **(B)** Synthesis of Xanthohumol D by Sugamoto et al.

Fu et al. reported the first synthesis of Xanthohumol D commenced from the Schenck ene reaction of intermediate **34**, which introduced the HMB groups. Then **35** was carried out in using catalytic amounts of *p*-TsOH leading to the target product Xanthohumol D (Fu et al., 2020) (Scheme 3A). The key intermediate **34** was prepared from **28** by using the method of Khupse et al. (Khupse and Erhardt, 2007). Sugamoto et al. (2020) also synthesized Xanthohumol D by using the same procedure employed for the synthesis of Xanthoangelol D from prenylated chalcone **(36)** (Scheme 3B).

### 2.5 Psorachalcone A (6)

#### 2.5.1 Isolation and biological activities

In 2001, ElSohly et al. isolated 2′,4′,4,2″-tetrahydroxy-3′-[3″-methylbut-3″-enyl]-chalcone **(6)** from an ethanol extract of the leaves of *Maclura tinctoria* (L.) Gaud, but did not give it a Latin name. Chalcone **6** showed inhibitory activity against *Candida albicans* (IC_50_ = 15 μg/ml) and *Cryptococcus neoformans* (IC_50_ = 7 μg/ml) (ElSohly et al., 2001; Nowakowska, 2007). Li et al. (2002) evaluated the fatty acid synthase inhibitory activity of chalcone **6**, and chalcone **6** exhibited marginal activity with IC_50_ of 46 μg/ml. Compound **6** was also isolated from the seeds of *Psoralea corylifolia*, and did not showed significant antibacterial activities against two pathogenic bacteria *Staphylococcus aureus* and *S. epidermidis* (MIC > 0.147 mM) (Yin et al., 2004). Ngadjui et al. (2005) isolated chalcone **6** from the twigs of *Dorstenia angusticornis* and *Dorstenia barteri* var. *subtriangularis*. Until 2005, Yu et al. isolated chalcone **6** and named it as Psorachalcone A (Yu et al., 2005; Xu et al., 2012). Zhai et al. evaluated the antiproliferative effects of synthetic Psorachalcone A against five cancer cells (PC-3, A375, PANC-1, A549 and MDA-MB-231), but no obvious inhibitory activity was observed (IC_50_ > 25 μM) (Zhai et al., 2019). Li et al. (2019) evaluated the antibacterial activities of synthetic Psorachalcone A against two Gram positive bacteria and two Gram negative bacteria, and Psorachalcone A showed slight activity towards Gram-positive bacteria (*Staphylococcus aureus*, MIC = 50 μg/ml; *Bacillus subtilis*, MIC = 25 μg/ml) but no obvious activity to Gram-negative bacteria (*Escherichia coli* and *Pseudomonas aeruginosa*, MIC > 200 μg/ml). In 2021, Psorachalcone A was isolated from the fruits of *Morus nigra* Linn., and did not exhibit obvious effect of inhibiting 3-phosphoglycerate dehydrogenase (Wang et al., 2021).

#### 2.5.2 Chemical synthesis

**SCHEME 4:**
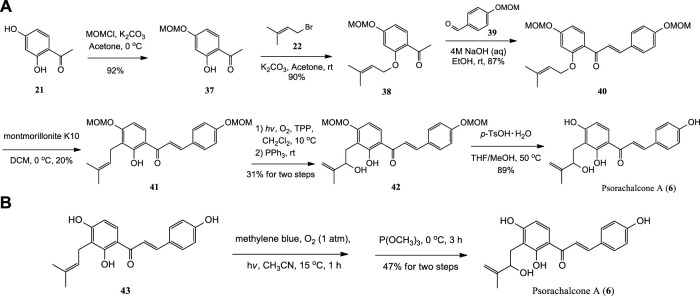
**(A)** Synthesis of Psorachalcone A by Zhai et al.; **(B)** Synthesis of Psorachalcone A by Sugamoto et al.

The first synthesis of Psorachalcone A and its new analogues were achieved from 2,4-dihydroxyacetophenone **(21)** through six steps by Zhai et al. (2019). Methoxymethyl (MOM) was used to protect the C4′-hydroxy group of 21 selectively. And MOM-protected **37** was prenylated with bromide **22** to afford **38**, which was further reacted with 39 to afford **40** by Claisen-Schmidt condensation. Then **40** was subjected to [1,3]-sigmatropic rearrangement, Schenck ene reaction and deprotection to obtain Psorachalcone A (Scheme 4A). Sugamoto et al. (2020) synthesized Psorachalcone A by using the same procedure employed for the synthesis of Xanthoangelol D from prenylated chalcone **(43)** (Scheme 4B).

### 2.6 Bartericin A and D (7, 10)

#### 2.6.1 Isolation and biological activities

Bartericin A and Bartericin D were isolated from the twigs of *Dorstenia barteri* var. *subtriangularis* successively, along with several other diprenylated chalcones (Ngameni et al., 2004; Ngadjui et al., 2005). (±)-Bartericin A obtained by the synthetic method was used to evaluate its antibacterial activity, and it showed moderate inhibitory activity against two Gram positive bacteria (*Staphylococcus aureus* and *Bacillus subtilis*, MIC = 25 μg/ml) (Fu et al., 2020).

#### 2.6.2 Chemical synthesis

**SCHEME 5:**
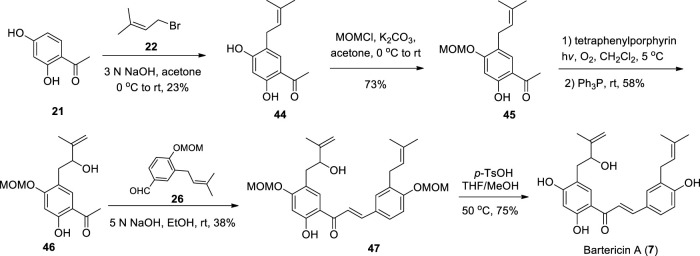
Synthesis of Bartericin A.

Fu et al. completed the first chemical synthesis of Bartericin A from the key intermediate **45** (Fu et al., 2020), and the preparation of **45** referred to the report of Dong et al. (Tan et al., 1999; Dong et al., 2007), which is similar to the synthetic steps of Paratocarpin E (Scheme 5). In addition, the chemical synthesis of Bartericin D has not been reported until now.

### 2.7 Angusticornin A and B (8, 9)

#### 2.7.1 Isolation and biological activities

Angusticornin A and B were first isolated from the twigs of *Dorstenia angusticornis* and *Dorstenia barteri* var. *subtriangularis* (Ngadjui et al., 2005; Simo et al., 2005). According to the report by Kuete et al., Angusticornin B didn′t exhibit obvious antimicrobial activity against a serials of Gram-negative multidrug-resistant bacteria, but increased significantly against *Escherichia coli* AG100A (MIC values, 64 vs. 16 mg/L) in the presence of the efflux pump inhibitor phenylalanine arginine β-naphthylamide (20 mg/L) (Kuete et al., 2011). Angusticornin A and B obtained by the synthetic method were used to evaluate their antibacterial activities, and only Angusticornin A showed moderate inhibitory activity against *Bacillus subtilis* (MIC = 25 μg/ml) (Li et al., 2019; Fu et al., 2020).

#### 2.7.2 Chemical synthesis

**SCHEME 6:**
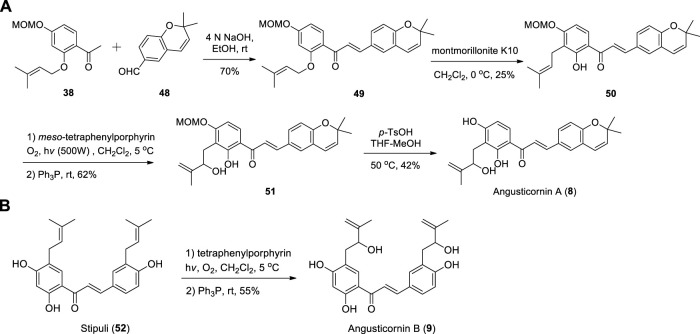
**(A)** Synthesis of Angusticornin A; **(B)** Synthesis of Angusticornin B.

Angusticornin A was synthesized from methyl ketone **38** and aldehyde **48** (Damodar et al., 2017) through Claisen-Schmidt condensation, [1,3]-sigmatropic rearrangement, Schenck ene reaction and deprotection (Li et al., 2019) (Scheme 6A). Angusticornin B was prepared from natural product Stipulin **(52)** by Schenck ene reaction (Scheme 6B) Fu et al. (2020).

### 2.8 2′,6′-dihydroxy-3′-(2-hydroxy-3-methyl-3-butenyl)-4′-methoxychalcone (11)

#### 2.8.1 Isolation and biological activities

Ren et al. isolated chalcone **11** from the whole plant of *Anaphalis lactea* (RenQi and Shi, 2008). The synthetic chalcone **11** was evaluated the antibacterial activities against two Gram positive bacteria (*Staphylococcus aureus* CMCC 26003, *Bacillus subtilis* CMCC(B) 63,501) and two Gram negative bacteria (*Escherichia coli* CMCC 44102, *Pseudomonas aeruginosa* CMCC(B) 10,104), but no obvious activity was observed in the four test strains (MIC > 200 μg/ml) (Fu et al., 2020).

#### 2.8.2 Chemical synthesis

**SCHEME 7:**
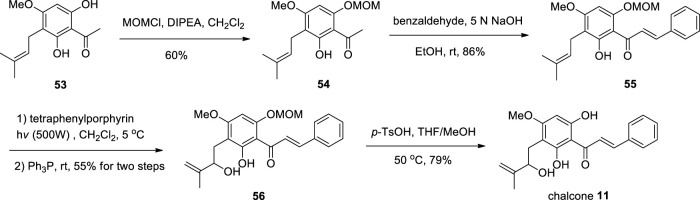
Synthesis of chalcone **11**.

According to Fu et al., methoxymethylation of compound **53** (Grayfer et al., 2016), and then subjected to Claisen-Schmidt condensation, Schenck ene reaction, and demethoxymethylation of **56** to obtain chalcone **11** (Fu et al., 2020) (Scheme 7).

### 2.9 Sanjuanolide (12)

#### 2.9.1 Isolation and biological activities

In 2016, Sanjuanolide was isolated from *Dalea frutescens* by Shaffer et al., and it exhibited slightly greater cytotoxic activities against PC-3 (IC_50_ = 11 ± 4 μM) and DU 145 (IC_50_ = 7 ± 3 μM) prostate cancer cell lines (Shaffer et al., 2016). Sanjuanolide was also isolated from the leaves and stem bark of *Artocarpus integer* in 2021 (Duong et al., 2021). Zhai et al. (2019) reported the anti-cancer activities of the synthetic Sanjuanolide against PC-3, A375, PANC-1, A549 and MDA-MB-231 cell lines, and Sanjuanolide showed moderate inhibitory activity against PC-3 (IC_50_ = 17.5 μM) and A375 (IC_50_ = 13.1 μM) cells. According to the report of Fang et al., (*R*)-Sanjuanolide efficiently inhibited the lipopolysaccharides-induced expression of tumor necrosis factor alpha and interleukin-6 (IC_50_ = 1.1 μM), but (*S*)-Sanjuanolide didn′t showed significant anti-inflammatory effect. Furthermore, (*R*)-Sanjuanolide effectively inhibited the mRNA expression of several inflammatory cytokines after the lipopolysaccharides challenge *in vitro* (Fang et al., 2019).

#### 2.9.2 Chemical synthesis

**SCHEME 8:**
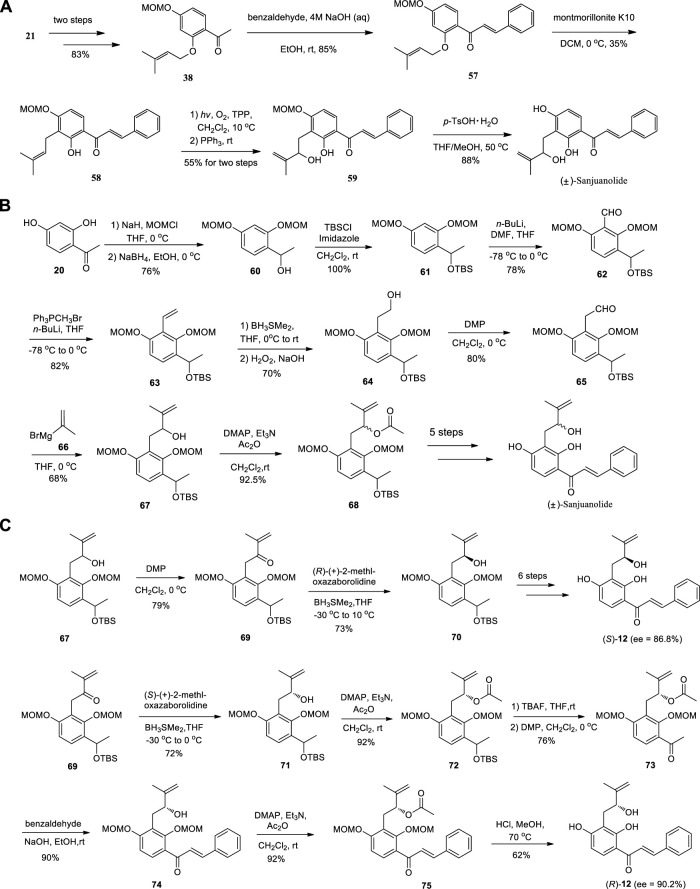
**(A)** Synthesis of (±)-Sanjuanolide by Zhai et al.; **(B)** Synthesis of (±)-Sanjuanolide by Fang et al.; **(C)** Synthesis of (*S*)-Sanjuanolide and (*R*)-Sanjuanolide by Fang et al.

Zhai et al. (2019) completed the total synthesis of (±)-Sanjuanolide from commercially available materials in seven steps (12% overall yield) (Scheme 8A), along with its seven new analogues. At the same time, Fang et al. completed the total synthesis of (±)-Sanjuanolide (Scheme 8B) in 15 steps with overall yield of 3.8%. In addition, (*S*)-Sanjuanolide and (*R*)-Sanjuanolide were also prepared in 17 steps with overall yields of 7.3% and 4.2%, respectively (Fang et al., 2019) (Scheme 8C).

### 2.10 2,2′,4,4′-tetrahydroxy-3-(2″-hydroxy-3″-methylbutyl-3″-alkenyl)chalcone (13)

Chalcone **13** was isolated from a *Morus alba* leaf by Yang et al., and it has a high free radical scavenging capacity while exhibits an IC_50_ of 21.6 μM against DPPH radicals (Yang and Jiang, 2021). In addition, no chemical synthesis of chalcone **13** has been reported so far.

## 3 Conclusion and outlook

Chalcone scaffolds, which is considered as the key bioactive precursors of plant flavonoids, have attracted more and more attention in medicinal chemistry and pharmacology. Herein, the isolation, chemical synthesis and biological activities of twelve natural chalcones and a chalcone-type compound bearing HMB group are reviewed. Although only a few dozen isolated or synthesized chalcones with HMB group have been reported, their various biological activities have aroused extensive interest of academic researchers, and it is believed that more and more natural or synthetic chalcones with HMB group will be presented in the further study. Furthermore, natural flavonoids with HMB group, which showed exciting biological activities, have also been reported by researchers in the past several decades, such as Ephedroidin and Dinklagin C (Lee et al., 1998; Pistelli et al., 1998; Xu et al., 2015; Owor et al., 2020). Moreover, further research on chalcones with HMB group might have much potential for drug discovery, especially as an adjuvant for a combination strategy between antibiotics and chalcones. And further studies on SAR, pharmacokinetics and toxicology are still needed, since the chemistry and biological importance of these biologically active compounds have not been systematically explored. Therefore, rational chemical derivatization of the natural chalcones and flavonoids bearing HMB group is necessary for further investigation of SAR, which play a key role in the screening of novel lead compounds.
